# Salivary Exosomes: From Waste to Promising Periodontitis Treatment

**DOI:** 10.3389/fphys.2021.798682

**Published:** 2022-01-05

**Authors:** Nik Nur Syazana Nik Mohamed Kamal, Wan Nazatul Shima Shahidan

**Affiliations:** School of Dental Sciences, Universiti Sains Malaysia, Health Campus, Kota Bharu, Malaysia

**Keywords:** saliva, exosome, periodontitis, waste, promising

## Abstract

Periodontitis is a chronic inflammatory condition that causes tooth loss by destroying the supporting components of the teeth. In most cases, it is difficult to diagnose early and results in severe phases of the disease. Given their endogenous origins, exosomes, which are rich in peptides, lipids, and nucleic acids, have emerged as a cell-free therapeutic approach with low immunogenicity and increased safety. Because the constituents of exosomes can be reprogrammed depending on disease states, exosomes are increasingly being evaluated to act as potential diagnostic biomarkers for dental disease, including periodontitis. Exosomes also have been demonstrated to be involved in inflammatory signal transmission and periodontitis progression *in vitro*, indicating that they could be used as therapeutic targets for periodontal regeneration. Nevertheless, a review on the involvement of salivary exosomes in periodontitis in impacting the successful diagnosis and treatment of periodontitis is still lacking in the literature. Thus, this review is intended to scrutinize recent advancements of salivary exosomes in periodontitis treatment. We summarize recent research reports on the emerging roles and characteristics of salivary exosomes, emphasizing the different expressions and changed biological roles of exosomes in periodontitis.

## Methodology and Delimitation

A PubMed database search on July 27, 2021 with the keywords “exosome AND periodontitis” resulted in a total of 86 articles. Only 81 articles remained after applying the “full-text” parameter. A total of 45 articles remained when a second parameter, “free full-text,” was added. Afterward, the articles were manually divided into two groups: original articles and “others” (not related to the topic, reviews, reports, editorials, commentaries, etc.). Here, we are focusing on original articles addressing salivary exosomes and periodontitis. Only six of the original articles on salivary exosomes and periodontitis were selected.

## Introduction

Periodontitis is a major public health concern with a high global prevalence (Papapanou et al., 2018). It is the most frequent osteolytic inflammatory severe disease, and it has been shown to have a detrimental effect on disease states like atherosclerosis, rheumatoid arthritis, and diabetes. The pathology is of an osteoimmune disorder defined by periodontal inflammation and subsequent destruction of the tooth-supporting tissue as alveolar bone, which is a leading cause of tooth loss in adults. Periodontitis is initiated by the accumulation of periodontal bacteria-associated biofilm, but it is not fully adequate to cause the disease since the host immune response is required for its development and progression (Yuki et al., 2021). The creation of high-impact diagnostic biomarkers that have a significant impact on clinical decision-making, patient outcomes, and healthcare providers is one of the goals of periodontology research (Ghallab, 2018). This could be accomplished by utilizing protein-containing exosomes.

Saliva has a variety of functions in the human body, including those that are crucial not only for the oral system and other bodily systems. Several research articles have been published in the last few years describing various salivary components and their distribution, confirming the biochemical composition and physiology of the proteins found in salivary fluids (Schenkels et al., 1995; Marotz et al., 2021). Many researchers have shown that exosomes may be found in human saliva and that they can be used to diagnose and investigate many diseases (Imai et al., 2021).

Exosomes are small vesicles (30–120 nm) that are secreted by all types of cultured cells and found in abundance in body fluids such as blood (Damanti et al., 2021), urine (Blijdorp et al., 2021), ascites (Cai et al., 2021), amniotic fluid (Bellio et al., 2020), and cultured medium of cell cultures (Ivica et al., 2020) including reticulocytes (Jiaqi et al., 2017); cytotoxic T lymphocytes (Chen et al., 2019); B lymphocytes (Calvo and Izquierdo, 2020); dendritic cells (Hosseini et al., 2021) and neoplastic intestinal epithelial cells (Scavo et al., 2020). These tiny vesicles play a key role in intercellular communication, both locally and systemically, allowing proteins, cytokines, and miRNA to be transferred between cells (Hergenreider et al., 2012). A review article comparing whether non-exosomal or exosomal miRNAs are more valid as biomarkers was recently published. Exosomes were chosen as the best origin for miRNAs used in biomarker studies (Nik Mohamed Kamal and Shahidan, 2020). Recently, exosomes have gained interest as a tool in regenerative medicine. They have been shown to be involved in the transmission of inflammatory signals and the development of periodontitis *in vitro*, indicating that they could be utilized as therapeutic targets for periodontal regeneration (Wang et al., 2020; Xin et al., 2020). Exosomes generated from dental pulp stem cells (DPSCs) have been found to minimize edema and enhance angiogenesis while suppressing inflammation (Pivoraite et al., 2015). Exosome-mediated dental pulp regeneration has only been demonstrated *in vitro* in the studies mentioned above. On the other hand, exosomes’ effects on the regeneration of dental pulp *in situ*, are little explored.

We evaluated over a decade of experience with salivary exosome, with an emphasis on the study of periodontitis. We compiled and updated the uses of salivary exosome as it relates to periodontitis from the literature and considered their physiological and clinical significance. We also examine their disease associations and potential clinical applications.

## Origin, Composition, and Potential Use of Extracellular Vesicles

**FIGURE 1:**
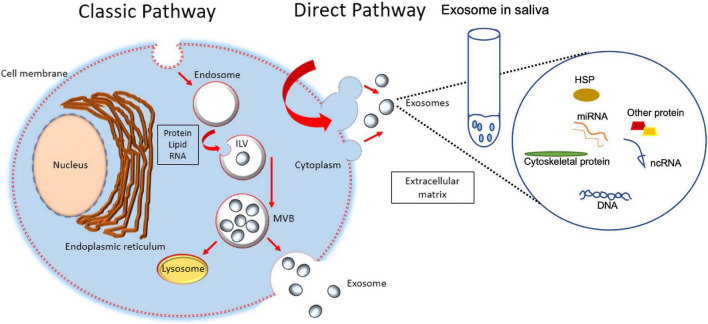
Formation of intraluminal vesicles (ILVs) within MVBs. Exosomes are derived from the multivesicular bodies (MVBs) which are known as late endosomes. MVBs are formed during the maturation of early endosomes into late endosomes with the accumulation of intraluminal vesicles. Upon maturation, MVBs are either destined for fusion with lysosomes where their contents will undergo lysosomal degradation, or with the plasma membrane where their contents are released into the extracellular space. The membrane of MVB fuses with the plasma membrane, resulting in the release of ILVs. When secreted, ILVs are called exosomes, release into saliva from submandibular gland cells and play a role in periodontal tissue and cells.

Exosomes, microvesicles, and apoptotic bodies are three types of vesicles secreted by cells, surrounded by lipid bilayer, and collectively known as extracellular vesicles (EVs) (Skotland et al., 2020). The vesicles can be distinguished by their sizes, biogenesis, and mechanism of release (Nik Mohamed Kamal and Shahidan, 2020). The diameter of the vesicles is recorded in the range of either 30–150 nm (exosomes), 100–1000 nm (microvesicles), or 1–5 μm (apoptotic bodies) (Rilla et al., 2019). The classical pathway for biogenesis of exosomes starts from the generation of endosomes from endocytoic activity of parent cells, leading to invagination of endosomal limiting membranes and formation of intraluminal vesicles (ILVs) that then mature into multivesicular bodies (MVBs). MVBs that are directed to plasma membranes are released into the extracellular environment as exosomes (Figure 1). Biogenesis of microvesicles is much simpler, where, upon stimulation, the outward parent cells’ membrane blebs. The blebs are then detached from parent cells as microvesicles. Biogenesis of apoptotic bodies starts from parent cells that are undergoing apoptosis (Skotland et al., 2020), leading to cell shrinkage and blebbing. The detached blebs are called apoptotic bodies (Rilla et al., 2019). Previous literature reported the detection of EVs from a variety of body fluids, e.g., bile (Nakashiki et al., 2021), saliva (Comfort et al., 2021), semen, blood (Vojtech et al., 2019), breast milk (Jiang et al., 2021), synovial fluid (Mustonen et al., 2021), urine (Blijdorp et al., 2021), and ascites (Bortot et al., 2021). Generally, these EVs are composed of nucleic acids [e.g., microRNAs (Nakashiki et al., 2021)], proteins (Bortot et al., 2021), lipids (Skotland et al., 2020), and signaling molecules (Vojtech et al., 2019). The contents can vary, depending on the parent cells, as well as the type of vesicles. For example, the existence of proteins in EVs could be influenced by the biogenesis of that particular EV, e.g., exosomes derived from the classical pathway might be enriched with protein associated with the endosomal pathway (e.g., Alix and TSG101) (Bruno et al., 2019). Up till now, many works of literature have been published describing EVs’ potential as biomarkers for diseases [e.g., asthma (Comfort et al., 2021), periodontitis (Kamal et al., 2020)], cargo for drug delivery [e.g., in treating wound healing, tissue repair, and regeneration (Rilla et al., 2019; Cao et al., 2021)], tools in understanding the membranes structure and mechanism for vesicular trafficking (Skotland et al., 2020), parts that function in the immune response system (e.g., source of self-antigens/forming immune complexes/autoantigen presentation) and as well as vesicles that contribute to the coagulation process (Turpin et al., 2016).

In other studies, the use of mesenchymal stromal cells (MSCs)-derived EV to treat chronic skin ulcers has offered a range of advantages, including accelerating healing and minimizing scar formation. This is the fact that the EV has immunosuppressive and immunomodulatory effects. They can also stimulate angiogenesis, proliferation, migration, and differentiation of the various cell types involved in skin regeneration (Casado-Díaz et al., 2020). In the case of using EVs as cargo for drug delivery systems, Villa et al. (2019) have described three different ways in their review paper. First, using tumor-derived EVs for targeting specific organs where EVs should be delivered to. By using tumor-derived EVs, the tumor-specific integrin expression pattern can be manipulated, which guarantees efficient organotropism (Sun et al., 2010; Hoshino et al., 2015). Secondly, using MSCs’ EVs to avoid oncogenic or immunogenic effects (Lai et al., 2019; Melzer et al., 2019). Thirdly, using light-induced hyperthermia to incorporate hollow gold nanoparticles into MSCs’ EVs to produce EVs that will be targeting specific cell types (Sancho-Albero et al., 2019). Interestingly, their potential application in the previously reported treatments opens the door for the design of new highly effective therapeutic strategies.

## Salivary Exosome

Although the mechanism of exosome biogenesis is still unclear, currently, there are two known ways of exosomal generation (Van der Pol et al., 2012; Gurung et al., 2021; Figure 1). Exosomes were reported to be released into the saliva either from the ductal or acinar cells. The salivary glands have been involved in constitutive-like secretory pathways that are involved in the secretion of vesicles such as exosomes. These secretory vesicles are derived directly from the *trans-*Golgi or involve elements of the endosomal-lysosomal trafficking pathway (Palanisamy et al., 2010). Exosomes can be shuttled from the systemic circulation into the oral cavity (Cheng et al., 2019).

Although the mechanisms of vesicle secretion in the blood are still unknown, findings from Marie-Pierre et al. (2005) gave strong evidence that exosomes can be secreted by all blood cells and retrieved in blood fluids. Since these EVs can cross the epithelial barriers, they may be essential for transporting components from the blood into saliva. Thus, in comparison with the other bodily fluids, salivary exosomes are probably a better and more accessible tool to examine the function of exosomes in the diagnosis and treatment of disease.

## Salivary Exosome in Periodontitis

Emerging functional and clinical applications of exosomes in human oral disease such as Oral disease, Oral squamous cell carcinoma, Primary Sjögren’s syndrome, Oral tissue regeneration, including periodontitis has been reported (Peng et al., 2020). The report discussed the role of exosomes in periodontitis, with increasing evidence that using natural nanostructured exosomes as a new disease treatment strategy is feasible. In early periodontal infection, periodontal ligament fibroblasts (PDLFs) are the predominant cell types that come into contact with pathogenic microbes. Human PDLF-derived exosomes enhance the expression of IL-6 and TNF-α in osteoblasts in response to lipopolysaccharide (LPS) stimuli, while simultaneously inhibiting the expression of osteogenesis-related elements such as collagen-I and osteoprotegerin and lowering alkaline phosphatase activity (Zhao et al., 2019). These findings suggest that LPS-pretreated PDLFs secrete exosomes, which cause inflammation and decrease osteogenic activity in osteoblasts. This is important to suggest that localized periodontal inflammation could influence bone remodeling by releasing exosomes. In the oral mechanical environment, PDLFs also help to maintain periodontal tissue homeostasis. When PDLFs were stimulated by cyclic stretch, they produced exosomes that inhibited the NF-κB signaling pathway, which suppressed IL-1 production in LPS-treated macrophages. PDLF cells in mechanical environments contribute to the maintenance of periodontal immune/inflammatory homeostasis by releasing exosomes (Wang et al., 2019). When they interact with their surrounding inflammatory milieu, periodontal ligament stem cells (PDLSCs) exhibit self-renewal ability and multipotency. Exosomes generated from LPS-stimulated PDLSCs contained more miR155 than its downstream target, Sirtuin1, which lowered T17 expression but enhanced Treg expression, relieving inflammation through the T17/Treg/miR1555p/Sirtuin1 regulatory network (Zheng et al., 2019). Periodontitis patients had higher levels of exosomal periodontal ligaments (PD-L1) mRNA in saliva than controls, and high levels of PD-L1 expression were linked to advanced stages of periodontitis. The level of salivary CD9 and CD81 exosomes, on the other hand, was reduced in periodontitis and was adversely linked with the disease state. According to the findings, an assay of exosome-based PD-L1 mRNA in saliva has the capacity to identify periodontitis from the healthy, and its levels correspond with periodontitis severity and stage (Tobón-Arroyave et al., 2019).

Kamal et al. (2020) demonstrated for the first time that miRNAs from plasma and salivary exosomes of chronic periodontitis (CP) patients were shown to be a possible diagnostic biomarker. When compared to prior CP miRNA studies, the profile revealed the most abundant number of miRNAs with a significant differential in expression. hsa-miR-125a-3p [*FC* = 2.03; *AUC* = 1; *r* = 0.91 (*p*-value = 0.02)] is worth additional investigation using salivary-exosomal samples. Because they were expressed considerably differently, had a good discriminatory value, and strongly correlated with the mean of periodontal pocket depth, these miRNAs can be concluded to be reliable candidates for the development of periodontitis biomarkers (Kamal et al., 2020).

The diagnostic potential of salivary small extracellular-associated (sEV) miRNAs in periodontal disease is being investigated for the first time by Han et al. (2020) in a pilot study. The ultimate goal was to evaluate the diagnostic utility of miRNAs extracted from sEVs to those extracted from total saliva in terms of differentiating between periodontal conditions. Three miRNAs (hsa-miR-140-5p, hsa-miR-146a-5p, and hsa-miR-628-5p) were found to be significantly upregulated (*p* 0.05) in salivary sEVs from periodontitis patients compared to healthy controls, suggesting that they could be potential periodontitis biomarkers. In microarray analysis of patients with CP, salivary hsa-miR-5571-5p, hsa-let-7f-5p, hsa-miR-99a-5p, hsa-miR-28-5p, and hsa-miR-320d expression was linked to periodontitis development. These salivary miRNAs could be novel biomarkers for periodontitis progression and monitoring them could lead to new periodontal diagnostics and precision therapy (Fujimori et al., 2021).

In various periodontal conditions, the pilot study of Han et al. (2021) gave important insight into the human global DNA methylation profiles of saliva sEVs and Gram-negative bacterial outer membrane vesicles (OMVs) (healthy, gingivitis, and periodontitis). The researchers discovered that periodontitis sEVs have considerably higher levels of LPS + OMVs, global 5mC methylation, and four periodontal pathogen OMVs (*Treponema denticola, Eikenella corrodens, Porphyromonas gingivalis, and Fusabacterium nucleatum*) than healthy sEVs.

## Salivary Exosomes’ Current and Future Potential in Periodontitis Treatment

The expression of PD-L1 mRNA in exosomes obtained from saliva of periodontitis patients was studied, as well as the clinical significance of salivary exosomes PD-L1 mRNA levels in the disease. The level of salivary exosomes PD-L1 mRNA in periodontitis patients differs significantly from non-periodontitis controls, according to one of the study’s key findings. Furthermore, a high amount of salivary exosomes PD-L1 was linked to advanced periodontitis, implying that it can represent disease progression. This is the first study to develop a method for detecting exosomal PD-L1 in saliva, as well as the first salivary exosomal biomarker for periodontitis (Yu et al., 2019). The specimens used in the majority of previous periodontitis biomarker studies have been gingival tissues or gingival crevicular fluids (GCF) (Kebschull et al., 2014). However, sample collection procedures for gingival tissues and GCF are challenging: gingival tissue biopsy requires invasive and limited tissue, while GCF sample collection requires sampling a minute amount of fluid on filter paper strips, which takes much longer.

Our previous study, which involved an easy, non-invasive, and quick collection of salivary specimens, demonstrated that a saliva-based assay overcomes the existing challenges. We also show that salivary exosomes may be used to extract miRNA, confirming the idea that exosome-derived samples protect miRNA from degradation (Kamal et al., 2020). According to recent studies, MSC-derived exosomes are increasingly being recognized as viable techniques to alleviate tissue injury and stimulate tissue regeneration in dental treatments, such as dental pulp regeneration, oral oncotherapy, and periodontal regeneration (Peng et al., 2020). To summarize, salivary exosomes may promote dental pulp regeneration by increasing the expression of specific proteins, promoting vascularization, modulating the interaction between epithelial and mesenchymal cells, and enhancing the abilities of dental pulp cells, all of which could be useful therapeutic methods in the future. Even though the current review focuses on salivary exosomes, some other study mimics the condition of salivary exosomes *in vitro* by collecting the exosome from the periodontal cell culture (Table 1). From Table 1, exosome research in periodontitis has only recently progressed, even though studies on exosomes and periodontitis have been ongoing for over a decade. Based on this progress, salivary exosomes showed potential as nano biomaterial that needs attention in the dental field.

**TABLE 1:** List progression of exosome study in periodontitis.

No.	Progression of exosome study in periodontitis	Source of exosome	Reference
1.	PDLF derived exosome help to maintain periodontal tissue homeostasis.	Culture media of PDLF cell	Wang et al., 2019
2.	PDLF derived exosome enhanced expression of IL-6 and TNF in response to LPS.	Culture media of PDLF cell	Zhao et al., 2019
3.	LPS-stimulated PDLSCs exosome relieve inflammation.	Culture media of PDLSCs cell	Zheng et al., 2019
4.	Exosome PD-L1 mRNA in saliva linked to advanced stage periodontitis.	Saliva	Tobón-Arroyave et al., 2019
5.	miRNAs from salivary exosomes of chronic periodontitis patients were shown to be a possible diagnostic biomarker.	Saliva	Kamal et al., 2020
6.	miRNA from saliva exosome could be reliable candidates for the development of periodontitis biomarker.	Saliva	Kamal et al., 2020
7.	The diagnostic potential of salivary small extracellular-associated miRNAs in periodontal disease is being investigated for the first time in the pilot study.	Saliva	Han et al., 2020
8.	In various periodontal conditions, the pilot study gave important insight into the human global DNA methylation profiles of saliva sEVs and Gram-negative bacterial outer membrane vesicles (OMVs) (healthy, gingivitis and periodontitis).	Saliva	Han et al., 2020
9.	Salivary exosome miRNA correlated with periodontitis progression.	Saliva	Fujimori et al., 2021

## Conclusion

Even though the role of salivary exosomes in periodontitis is very limited, based on growing evidence, exosomes may play a significant role in the regulation of periodontitis. Exosomes derived from saliva act as essential promoters in periodontal regenerators and periodontitis biomarkers, which have been a research focus all along. Exosomes’ effects on oral diseases, such as periodontitis have received increasing attention in recent years, giving us a better understanding of the functions that exosomes play in oral diseases. Furthermore, saliva exosomes are economical, carrying numerous biological components that have a lot of promise for assisting clinical diagnosis and determining prognosis.

## Author Contributions

NNS wrote the first draft of the manuscript. WNS revised the manuscript. Both authors approved the final version of the manuscript and agreed to be accountable for all aspects of the work.

## Conflict of Interest

The authors declare that the research was conducted in the absence of any commercial or financial relationships that could be construed as a potential conflict of interest.

## Publisher’s Note

All claims expressed in this article are solely those of the authors and do not necessarily represent those of their affiliated organizations, or those of the publisher, the editors and the reviewers. Any product that may be evaluated in this article, or claim that may be made by its manufacturer, is not guaranteed or endorsed by the publisher.

## References

[B1] Bellio M. A. Abdullah Z. Steward D. Khan A. Xu X. Shapiro G. C. (2020). MicroRNA sequencing of amniotic fluid derived exosome cargo reveals a therapeutic potential for the treatment of osteoarthritis. *Cytotherapy* 22:S47. 10.1016/j.jcyt.2020.03.054

[B2] Blijdorp C. J. Tutakhel O. A. Hartjes T. A. van den Bosch T. P. van Heugten M. H. Rigalli J. P. (2021). Comparing approaches to normalize, quantify, and characterize urinary extracellular vesicles. *J. Am. Soc. Nephrol.* 32 1210–1226. 10.1681/ASN.2020081142 33782168 PMC8259679

[B3] Bortot B. Apollonio M. Rampazzo E. Valle F. Brucale M. Ridolfi A. (2021). Malignant-ascites-derived small extracellular vesicles in advanced ovarian cancer patients: insights into the dynamics of the extracellular matrix. *Mole. Oncol.* 6:13110. 10.1002/1878-0261.13110 34614287 PMC8637559

[B4] Bruno S. Chiabotto G. Favaro E. Deregibus M. C. Camussi G. (2019). Role of extracellular vesicles in stem cell biology. *Am. J. Physiol. Cell Physiol.* 317 C303–C313. 10.1152/ajpcell.00129.2019 31091143 PMC6732418

[B5] Cai J. Gong L. Li G. Guo J. Yi X. Wang Z. (2021). Exosomes in ovarian cancer ascites promote epithelial–mesenchymal transition of ovarian cancer cells by delivery of miR-6780b-5p. *Cell Death Dis.* 12 1–17. 10.1038/s41419-021-03490-5 33627627 PMC7904844

[B6] Calvo V. Izquierdo M. (2020). Inducible polarized secretion of exosomes in T and B lymphocytes. *Int. J. Mol. Sci.* 21:2631. 10.3390/ijms21072631 32290050 PMC7177964

[B7] Cao Z. Wu Y. Yu L. Zou L. Yang L. Lin S. (2021). Exosomal miR-335 derived from mature dendritic cells enhanced mesenchymal stem cell-mediated bone regeneration of bone defects in athymic rats. *Mole. Med.* 27 1–13. 10.1186/s10020-021-00268-5 33637046 PMC7913386

[B8] Casado-Díaz A. Quesada-Gómez J. M. Dorado G. (2020). Extracellular Vesicles Derived From Mesenchymal Stem Cells (MSC) in Regenerative Medicine: Applications in Skin Wound Healing. *Front. Bioeng. Biotechnol.* 8:146. 10.3389/fbioe.2020.00146 32195233 PMC7062641

[B9] Chen L. Huang H. Zhang W. Ding F. Fan Z. Zeng Z. (2019). Exosomes derived from t regulatory cells suppress CD8+ cytotoxic T lymphocyte proliferation and prolong liver allograft survival. *Med. Sci. Mon.* 25 4877–4884. 10.12659/MSM.917058 31258152 PMC6618337

[B10] Cheng J. Nonaka T. Wong D. (2019). Salivary exosomes as nanocarriers for cancer biomarker delivery. *Materials* 12:654. 10.3390/ma12040654 30795593 PMC6416587

[B11] Comfort N. Bloomquist T. R. Shephard A. P. Petty C. R. Cunningham A. Hauptman M. (2021). Isolation and characterization of extracellular vesicles in saliva of children with asthma. *Extracell. Ves. Circul. Nucleic Acids* 2 29–48. 10.20517/evcna.2020.09 34368811 PMC8340923

[B12] Damanti C. C. Gaffo E. Lovisa F. Garbin A. Di Battista P. Gallingani I. (2021). miR-26a-5p as a reference to normalize microRNA qRT-PCR levels in plasma exosomes of pediatric hematological malignancies. *Cells* 10:101. 10.3390/cells10010101 33429910 PMC7827902

[B13] Fujimori K. Yoneda T. Tomofuji T. Ekuni D. Azuma T. Maruyama T. (2021). Detection of salivary miRNAs that predict chronic periodontitis progression: A Cohort Study. *Int. J. Environ. Res. Public Health* 18:8010. 10.3390/ijerph18158010 34360304 PMC8345340

[B14] Ghallab N. A. (2018). Diagnostic potential and future directions of biomarkers in gingival crevicular fluid and saliva of periodontal diseases: review of the current evidence. *Arch. Oral. Biol.* 87 115–124. 10.1016/j.archoralbio.2017.12.022 29288920

[B15] Gurung S. Perocheau D. Touramanidou L. Baruteau J. (2021). The exosome journey: from biogenesis to uptake and intracellular signalling. *Cell Comm. Signal.* 19 1–19. 10.1186/s12964-021-00730-1 33892745 PMC8063428

[B16] Han P. Bartold P. M. Salomon C. Ivanovski S. (2020). Salivary small extracellular vesicles associated miRNAs in periodontal status- A pilot study. *Int. J. Mol. Sci.* 21:2809. 10.3390/ijms21082809 32316600 PMC7215885

[B17] Han P. Bartold P. M. Salomon C. Ivanovski S. (2021). Salivary outer membrane vesicles and DNA methylation of small extracellular vesicles as biomarkers for periodontal status: A pilot study. *Int. J. Mol. Sci*. 22:2423. 10.3390/ijms22052423 33670900 PMC7957785

[B18] Hergenreider E. Heydt S. Treguer K. Boettger T. Horrevoets A. J. Zeiher A. M. (2012). Atheroprotective communication between endothelial cells and smooth muscle cells through miRNAs. *Nat. Cell Biol.* 14 249–256. 10.1038/ncb2441 22327366

[B19] Hoshino A. Costa-Silva B. Shen T. L. Rodrigues G. Hashimoto A. Tesic Mark M. (2015). Tumour exosome integrins determine organotropic metastasis. *Nature* 527 329–335. 10.1038/nature15756 26524530 PMC4788391

[B20] Hosseini R. Asef-Kabiri L. Yousefi H. (2021). The roles of tumor-derived exosomes in altered differentiation, maturation and function of dendritic cells. *Mol. Cancer* 20:83. 10.1186/s12943-021-01376-w 34078376 PMC8170799

[B21] Imai A. Oka S. Susuga M. Tsutsui N. Haga-Tsujimura M. Saitoh E. (2021). Comprehensive analysis and comparison of proteins in salivary exosomes of climacteric and adolescent females. *Odontology* 109 82–102. 10.1007/s10266-020-00538-4 32681298

[B22] Ivica A. Ghayor C. Zehnder M. Valdec S. Weber F. E. (2020). Pulp-derived exosomes in a fibrin-based regenerative root filling material. *J. Clin. Med.* 9:491. 10.3390/jcm9020491 32054086 PMC7074310

[B23] Jiang X. You L. Zhang Z. Cui X. Zhong H. Sun X. (2021). Biological properties of milk-derived extracellular vesicles and their physiological functions in infant. *Front. Cell Dev. Biol.* 9:1591. 10.3389/fcell.2021.693534 34249944 PMC8267587

[B24] Jiaqi W. Xuang S. Zhao J. Yang Y. Cai X. Xu J. (2017). Exosomes: A Novel Strategy for Treatment and Prevention of Diseases. *Front. Pharm.* 8:300. 10.3389/fphar.2017.00300 28659795 PMC5468768

[B25] Kamal N. N. S. N. M. Awang R. A. R. Mohamad S. Shahidan W. N. S. (2020). Plasma-and saliva exosome profile reveals a distinct microRNA signature in chronic periodontitis. *Front. Physiol.* 2020:11. 10.3389/fphys.2020.587381 33329037 PMC7733931

[B26] Kebschull M. Demmer R. T. Grün B. Guarnieri P. Pavlidis P. Papapanou P. N. (2014). Gingival tissue transcriptomes identify distinct periodontitis phenotypes. *J Dent Res* 93 459–468. 10.1177/0022034514527288 24646639 PMC3988627

[B27] Lai P. Weng J. Guo L. Chen X. Du X. (2019). Novel insights into MSC-EVs therapy for immune diseases. *Biomark. Res*. 7:6. 10.1186/s40364-019-0156-0 30923617 PMC6423844

[B28] Marie-Pierre C. Danielle L. Claude V. S. Graça R. Christian B. (2005). Exosomal-like vesicles are present in human blood plasma. *Internat. Immunol.* 17 879–887. 10.1093/intimm/dxh267 15908444

[B29] Marotz C. Morton J. T. Navarro P. Coker J. Belda-Ferre P. Knight R. (2021). Quantifying live microbial load in human saliva samples over time reveals stable composition and dynamic load. *Msystems* 6 e1182–e1120. 10.1128/mSystems.01182-20 33594005 PMC8561659

[B30] Melzer C. Rehn V. Yang Y. Bähre H. von, der Ohe J. (2019). Taxol-loaded MSC-derived exosomes provide a therapeutic vehicle to target metastatic breast cancer and other carcinoma cells. *Cancers* 11:798. 10.3390/cancers11060798 31181850 PMC6627807

[B31] Mustonen A. M. Capra J. Rilla K. Lehenkari P. Oikari S. Kaariainen T. (2021). Characterization of hyaluronan-coated extracellular vesicles in synovial fluid of patients with osteoarthritis and rheumatoid arthritis. *BMC Musculoskel. Dis.* 22:1–11. 10.1186/s12891-021-04115-w 33676459 PMC7937210

[B32] Nakashiki S. Miuma S. Mishima H. Masumoto H. Hidaka M. Soyama A. (2021). Bile extracellular vesicles from end-stage liver disease patients show altered microRNA content. *Hepatol. Internat.* 2021 1–10. 10.1007/s12072-021-10196-5 34076850

[B33] Nik Mohamed Kamal N. N. S. B. Shahidan W. N. S. (2020). Non-Exosomal and Exosomal Circulatory MicroRNAs: Which Are More Valid as Biomarkers? *Front. Pharm.* 10:1500. 10.3389/fphar.2019.01500 32038230 PMC6984169

[B34] Palanisamy V. Sharma S. Deshpande A. Zhou H. Gimzewski J. Wong D. T. (2010). Nanostructural and transcriptomic analyses of human saliva derived exosomes. *PLoS One* 5:e8577. 10.1371/journal.pone.0008577 20052414 PMC2797607

[B35] Papapanou P. N. Sanz M. Buduneli N. Dietrich T. Feres M. Fine D. H. (2018). Periodontitis: Consensus report of workgroup 2 of the 2017 world workshop on the classification of periodontal and peri-Implant diseases and conditions. *J. Period.* 89 S173–S182. 10.1002/JPER.17-0721 29926951

[B36] Peng Q. Yang J. Y. Zhou G. (2020). Emerging functions and clinical applications of exosomes in human oral diseases. *Cell Biosci*. 10:68. 10.1186/s13578-020-00424-0 32489584 PMC7245751

[B37] Pivoraite U. Jarmalaviciute A. Tunaitis V. Ramanauskaite G. Vaitkuviene A. Kaseta V. (2015). Exosomes from human dental pulp stem cells suppress carrageenan-induced acute inflammation in mice. *Inflammation* 38 1933–1941. 10.1007/s10753-015-0173-6 25903966

[B38] Rilla K. Mustonen A. M. Arasu U. T. Härkönen K. Matilainen J. Nieminen P. (2019). Extracellular vesicles are integral and functional components of the extracellular matrix. *Matrix Biol.* 75 201–219. 10.1016/j.matbio.2017.10.003 29066152

[B39] Sancho-Albero M. Navascués N. Mendoza G. Sebastián V. Arruebo M. Martín-Duque P. (2019). Exosome origin determines cell targeting and the transfer of therapeutic nanoparticles towards target cells. *J. Nanobiotechnol.* 17:16. 10.1186/s12951-018-0437-z 30683120 PMC6346572

[B40] Scavo M. P. Rizzi F. Depalo N. Fanizza E. Ingrosso C. Curri M. L. (2020). A possible role of FZD10 delivering exosomes derived from colon cancers cell lines in inducing activation of epithelial–mesenchymal transition in normal colon epithelial cell line. *Internat. J. Mole. Sci.* 21:6705. 10.3390/ijms21186705 32933173 PMC7555665

[B41] Schenkels L. C. P. M. Veerman E. C. I. Amerongen A. V. N. (1995). Biochemical composition of human saliva in relation to other mucosal fluids. *Crit. Rev. Oral Biol. Med.* 6 161–175. 7548622 10.1177/10454411950060020501

[B42] Skotland T. Sagini K. Sandvig K. Lorente A. (2020). An emerging focus on lipids in extracellular vesicles. *Adv. Drug Del. Rev.* 159 308–321. 10.1016/j.addr.2020.03.002 32151658

[B43] Sun D. Zhuang X. Xiang X. Liu Y. Zhang S. Liu C. (2010). A novel nanoparticle drug delivery system: The anti-inflammatory activity of curcumin is enhanced when encapsulated in exosomes. *Mol. Ther*. 18 1606–1614. 10.1038/mt.2010.105 20571541 PMC2956928

[B44] Tobón-Arroyave S. I. Celis-Mejía N. Córdoba-Hidalgo M. P. Isaza-Guzmán D. M. (2019). Decreased salivary concentration of CD9 and CD81 exosomerelated tetraspanins may be associated with the periodontal clinical status. *J. Clin. Periodontol.* 46 470–480. 10.1111/jcpe.13099 30825338

[B45] Turpin D. Truchetet M. E. Faustin B. Augusto J. F. Contin-Bordes C. Brisson A. (2016). Role of extracellular vesicles in autoimmune diseases. *Autoimm. Rev.* 15 174–183. 10.1016/j.autrev.2015.11.004 26554931

[B46] Van der Pol E. Böing A. N. Harrison P. Sturk A. Nieuwland R. (2012). Classification, functions, and clinical relevance of extracellular vesicles. *Pharmacolog. Rev.* 64 676–705. 10.1124/pr.112.005983 22722893

[B47] Villa F. Quarto R. Tasso R. (2019). Extracellular vesicles as natural, safe and efficient drug delivery systems. *Pharmaceutics* 11:557. 10.3390/pharmaceutics11110557 PMC6920944 31661862

[B48] Vojtech L. Zhang M. Davé V. Levy C. Hughes S. M. Wang R. (2019). Extracellular vesicles in human semen modulate antigen-presenting cell function and decrease downstream antiviral T cell responses. *PLo*S *One* 14:e0223901. 10.1371/journal.pone.0223901 PMC6797208 31622420

[B49] Wang R. Ji Q. Meng C. Liu H. Fan C. Lipkind S. (2020). Role of gingival mesenchymal stem cell exosomes in macrophage polarization under inflammatory conditions. *Int. Immunopharmacol.* 81:106030. 10.1016/j.intimp.2019.106030 31796385

[B50] Wang Z. Maruyama K. Sakisaka Y. Suzuki S. Tada H. Suto M. (2019). Cyclic stretch force induces periodontal ligament cells to secrete exosomes that suppress IL-1β production through the inhibition of the NF-κB signalling pathway in macrophages. *Front. Immunol.* 10:1310. 10.3389/fimmu.2019.01310 PMC6595474 31281309

[B51] Xin X. Shuang H. Zhi L. Zubing L. (2020). Emerging role of exosomes in craniofacial and dental applications. *Theranostics* 10 8648–8664. 32754269 10.7150/thno.48291 PMC7392016

[B52] Yu J. Lin Y. Xiong X. Li K. Yao Z. Dong H. (2019). Detection of exosomal PD-L1 RNA in saliva of patients with periodontitis. *Front. Genet.* 10:202. 10.3389/fgene.2019.00202 PMC6426748 30923536

[B53] Yuki N. Takao F. Qunzhou Z. Terukazu S. Takanori S. Xiaoxing K. (2021). Exosomes from TNF-α-treated human gingiva-derived MSCs enhance M2 macrophage polarization and inhibit periodontal bone loss. *Acta Biomat.* 122 306–324. 10.1016/j.actbio.2020.12.046 PMC7897289 33359765

[B54] Zhao M. Dai W. Wang H. Xue C. Feng J. He Y. (2019). Periodontal ligament fibroblasts regulate osteoblasts by exosome secretion induced by infammatory stimuli. *Arch. Oral. Biol.* 105 27–34. 31247478 10.1016/j.archoralbio.2019.06.002

[B55] Zheng Y. Dong C. Yang J. Jin Y. Zheng W. Zhou Q. (2019). Exosomal microRNA-155-5p from PDLSCs regulated Th17/Treg balance by targeting sirtuin-1 in chronic periodontitis. *J. Cell. Physiol.* 234:1. 10.1002/jcp.28671 31016751

